# Metabolic Profiling of Aromatic Compounds

**DOI:** 10.3390/metabo14020107

**Published:** 2024-02-05

**Authors:** Alisa K. Pautova

**Affiliations:** Federal Research and Clinical Center of Intensive Care Medicine and Rehabilitology, 25-2 Petrovka Str., 107031 Moscow, Russia; alicepau@mail.ru; Tel.: +7-905-773-89-81

Metabolic profiling is a powerful modern tool in searching for novel biomarkers and indicators of normal or pathological processes in the body. The metabolic profiling of an object is the result of a nontargeted analysis, which is usually aimed at the identification of as many individual metabolites as possible using chromatography–mass spectrometry or nuclear magnetic resonance (NMR) spectroscopy and statistical data processing. This reveals the metabolites that are characteristic for specific conditions or pathologies. Hence, metabolic profiling is a complex, time-consuming, and expensive procedure that involves the coordinated work of specialists from different fields. However, its main result is aimed at a panel of the most promising biomarkers that could be subsequently analyzed in routine clinical practice using available and relatively cheap technology.

The content of low-molecular-weight compounds in biological samples reflects a portrait of changes occurring at the cellular, organ, and system levels of the body. Hence, the biological objects for metabolic profiling can be blood plasma or serum, urine, cerebrospinal or other types of bodily fluids, feces, exhaled breath, tissues, cells, or some specific objects. This Special Issue contains papers in which metabolic profiling was studied in blood serum (contributions 1–3), drainage fluid (contribution 1), exhaled breath (contribution 4), and HepG2 cells (contribution 5).

Chromatography–mass spectrometry methods occupy a leading position in metabolic profiling studies. Ultra-high-performance liquid chromatography coupled to quadrupole time-of-flight tandem mass spectrometry (UPLC-QTOF) and gas chromatography-quadrupole (GC-MS) or time-of-flight mass spectrometry (GC-TOF) are the most advanced and appropriate analytical platforms for metabolic profiling. Both techniques provide the accurate mass-to-charge ratio of a compound or its fragments, and special databases are used list candidates from the mass spectra data obtained [1]. All studies in the current Special Issue applied chromatography–mass spectrometry-based analytical platforms.

The articles in the current Special Issue unraveled the metabolic profiles of aromatic compounds in healthy people (contribution 2) and elite athletes (contribution 3) together with metabolic changes following severe oncological diseases (contributions 1, 4, 5). The topics covered in this Special Issue are devoted to actual and highly substantial issues of medicine. A literature search by querying “metabolic profiling” in PubMed increased from 3800 in 2014 to 11,600 in 2022 and 2023 (data on 11 January 2024). Thus, metabolic profiling is widely used in studying cancer as it can track individual changes in tumor metabolism and gauge treatment response across time, measuring medication efficacy and monitoring drug resistance [2]. Searching for “metabolic profiling & cancer” in PubMed resulted in an approximately equal number of 2100 papers over the last 3 years (data on 12 January 2024). Further in this text, some selected works from this list will be briefly presented, demonstrating the breadth of application of metabolic profiling for diagnosing a wide variety of cancer types. Also, the potential biomarkers of aromatic structures will be listed.

The tissue metabolic profiling of colorectal cancer using a UPLC-QTOF method revealed metabolic alteration in the content of 14 metabolites, including serotonin, 5-hydroxytryptophol, 5-hydroxyindoleacetate, tryptophan, kynurenine, and long chain acylcarnitines [3]. A urinary metabolic profiling analysis of colorectal cancer using GC-TOF resulted in 15 significant metabolites, including tryptophan and tyrosine [4]. The tissue metabolic profiling of human gastric cancer, assessed with NMR, revealed 48 endogenous distinguishing metabolites, including phenylacetylglutamine, tyrosine, and tryptophane [5]. The urinary metabolic profiling of bladder cancer using NMR and two methods of high-resolution nanoparticle-based laser desorption/ionization mass spectrometry (LDI-MS) led to the identification of five potentially robust metabolic indicators, which included 4-hydroxyphenylacetate, Hippurate, and trigonelline [6]. The fecal metabolic profiling of breast cancer using NMR revealed 27 potential biomarkers, which reflected the influence of chemotherapy. They were amino, short-chain fatty, and Krebs cycle acids, as well as some compounds, namely phenylacetate and 3,4-dihydroxybenzeneacetate [7]. The large-scale profiling of metabolic dysregulation in epithelial ovarian cancer assessed by UPLC-QTOF proposed a list 53 metabolites, including 3-indolepropionic acid, 5-hydroxyindoleacetaldehyde, and 4-hydroxyphenyllactic acid [8]. The study of serum and urine metabolomics using NMR revealed a distinct diagnostic model for cancer cachexia using 15 metabolites, including phenylacetate and tryptophan [9]. HepG2 cells are used as a model for studying liver cancer [10], and the articles describing its metabolic profiling were accumalated by Kiseleva et al. in the only Review in this Special Issue (contribution 5).

A blood-based metabolomic signature assessed using UPLC-QTOF provided a three-marker microbial-related metabolite panel to assess the 5-year risk for pancreatic cancer; this panel included indoleacrylic acid [11]. Differences in the serum metabolic profile, including the level of microbial p-hydroxyphenyllactic acid, detected by GC-MS were found between patients with pancreatic cancer and healthy people and are described in a research article by Getsina et al. Additionally, the metabolic profile of drainage fluid was described and differences in the drainage of patients undergoing chemotherapy before surgery were revealed (contribution 1).

A serum metabolic profiling study of lung cancer using UPLC-QTOF resulted in 27 differential metabolites [12]. The metabolic profiling of potential lung cancer biomarkers using bronchoalveolar lavage fluid and integrated direct infusion/gas chromatography mass spectrometry techniques revealed 42 altered metabolites, including benzoic acid [13]. The metabolic profiling of volatile organic compounds from patients and cell lines for the validation of lung cancer biomarkers by proton-transfer-reaction mass spectrometry resulted in three main metabolites: acetaldehyde, acetone, and 1(2)-propanol [14]. Diagnostic models for lung cancer using the profile of volatile organic compounds obtained by GC-MS in exhaled breath were developed in the research study by Temerdashev et al. in the current Special Issue and included benzaldehyde, ethylbenzene, and propylbenzene (contribution 4).

Much attention is paid to studying central nervous system diseases using metabolic profiling [15]. The study of plasma metabolic profiles in patients with Parkinson’s disease using UPLC-QTOF resulted in a metabolite panel of four biomarker candidates, including two fatty acids, 3-indolelactic acid, and phenylacetyl-glutamine [16]. Plasma metabolic alterations in multiple sclerosis and the impact of vitamin D supplementation using UPLC-MS/MS were studied in a clinical trial that resulted in a list of candidate biomarkers, including phenylpyruvate [17]. The metabolomic signature of symptom dimensions in major depression assessed using GC-TOF revealed the altered profile of various metabolites, including phenylalanine, indoxyl sulfate, p-cresol sulfate, and benzyl alcohol [18]. Disturbances in fecal metabolism in post-stroke depression were detected using GC-MS and included the alteration of L-kynurenine, 5-methoxytryptamine, tyramine, and phenylacetic acid [19]. The metabolic profiling of serum samples of patients with spinocerebellar ataxia 3, also known as Machado–Joseph disease, using GC-MS and UPLC-MS revealed four potential biomarkers, including L-tryptophan [20]. The metabolic profiling of cerebrospinal fluid using GC-MS methods, obtained from patients with various types of central nervous system diseases, including inflammatory demyelinating, neurodegenerative, oncological, infectious, mental, genetic, vascular, and epileptic diseases, resulted in a panel of metabolites, including metabolites of tryptophan, arachidonic acid, and glucose; amino, polyunsaturated fatty, and other organic acids; and neuroactive steroids. They are accumulated in a review [21].

When describing lists of the metabolites that are potential biomarkers of the above-described pathologies, it was not by chance that metabolites with an aromatic structure were highlighted. Most of these compounds are aromatic α-amino acids (AAAs) tyrosine, phenylalanine, and tryptophane, or metabolites of these acids. In humans, AAAs are used to synthesize proteins or a variety of biologically active compounds, such as neurotransmitters and hormones, that are essential for maintaining normal biological functions [22]. Also, there are a number of aromatic metabolites that are known to be important microbial metabolites of these AAAs. They are metabolites of phenylalanine (phenylacetic, phenyllactic, and phenylpropionic acids), tyrosine (4-hydroxyphenylpropionic, 4-hydroxyphenylacetate, and 4-hydroxyphenyllactate acids), and tryptophan (indolepropionic, indoleacetic, indolelactic, and indolepropionic acids) [23]. These metabolites were detected in healthy people [24], and a validated method for their simultaneous determination was developed by Sobolev et al. in one of the articles in the current Special Issue (contribution 2). The reference values of these metabolites can be used in further clinical studies.

The phenomenon that aromatic metabolites of AAAs, including those of microbial origin, were reported to be potential biomarkers of various diseases illustrates the enormous and incompletely studied impact of the microbial function of the human gut microbiota on host health [25,26]. The presence of the gut microbiota–brain axis [27] and some other interactions (the gut microbiome–lung axis [28] and the gut microbiome–heart axis [29]) affecting patients in various conditions, including those in critical states, were postulated in the modern scientific community [30]. Thus, the aim of this Special Issue, which was to accumulate the results of different studies revealing the diversity in the properties of aromatic compounds and their role in metabolic pathways, was achieved and will allow scientists to learn more about this actual topic.

## Data Availability

Not applicable.
